# Advances in alternative splicing identification: deep learning and pantranscriptome

**DOI:** 10.3389/fpls.2023.1232466

**Published:** 2023-09-18

**Authors:** Fei Shen, Chenyang Hu, Xin Huang, Hao He, Deng Yang, Jirong Zhao, Xiaozeng Yang

**Affiliations:** ^1^ Institute of Biotechnology, Beijing Academy of Agriculture and Forestry Sciences, Beijing, China; ^2^ Shanxi Key Lab of Chinese Jujube, College of Life Science, Yan’an University, Yan’an, Shanxi, China

**Keywords:** alternative splicing, RNA-seq, Iso-seq, detection algorithm, deep learning, pantranscriptome

## Abstract

In plants, alternative splicing is a crucial mechanism for regulating gene expression at the post-transcriptional level, which leads to diverse proteins by generating multiple mature mRNA isoforms and diversify the gene regulation. Due to the complexity and variability of this process, accurate identification of splicing events is a vital step in studying alternative splicing. This article presents the application of alternative splicing algorithms with or without reference genomes in plants, as well as the integration of advanced deep learning techniques for improved detection accuracy. In addition, we also discuss alternative splicing studies in the pan-genomic background and the usefulness of integrated strategies for fully profiling alternative splicing.

## 1 The alternative splicing event in plants

### 1.1 Definition and classification of alternative splicing

Alternative splicing (AS) is a crucial mechanism for gene expression regulation, which entails the selection of different splice sites, removal of introns, and subsequent combine various exons to generate multiple mature mRNA isoforms in plants (Barbazuk et al., 2008). Plants generate extensive AS to increase the diversity of their transcriptomes, especially faced with complex environmental changes (Nilsen and Graveley, 2010; Szakonyi and Duque, 2018; Jia et al., 2022; Lam et al., 2022). There are several types of AS events in plants, including exon skipping (ES), intron retention (IR), alternative 5′ splice site (AE5′), alternative 3′ splice site (AE3′), mutually exclusive alternate exon splicing (MEE), alternative first exon (AFE), and alternative last exon (ALE) (Filichkin et al., 2010; E et al., 2013; Chen et al., 2020b). Among them, IR is the predominant type (Syed et al., 2012; Zhu et al., 2017).

### 1.2 Generation of alternative splicing

The spliceosome is a large ribonucleoprotein complex that interacts with various trans-acting factors and is involved in controlling AS in plants (Will and Luhrmann, 2010; Ule and Blencowe, 2019; Liu et al., 2021; Jia et al., 2022). The U2 and U12 spliceosomal RNA are the focus RNAof most studies on the spliceosome (Hartmann, 2007; Reddy et al., 2012; Zhang et al., 2020). The spliceosome splices intron-exon junction sites, which are characterized by the conserved 5′-GT sequence and AG-3′ sequence. Non-snRNA (small nuclear RNA) splicing factors, such as serine/arginine-rich proteins and heterogeneous ribonucleoproteins, are known to facilitate the localization of splicing enhancers and inhibitors, thereby regulating the selection of splice sites (Geuens et al., 2016; Jeong, 2017; Chen et al., 2020a). Pre-mRNA undergoes two consecutive reactions to complete the splicing process: (i) introns form a unique chain-like structure; (ii) intron are rapidly degraded as a chain-like structure, and exons at the left and right ends are joined by phosphodiester bonds, achieving intron excision and exon joining (Black, 2003; Wan et al., 2019).

### 1.3 Functionality of alternative splicing

AS plays a crucial role in regulating plant growth, development and responses to abiotic stresses. AS generally occurs during seed germination, plant growth, and flowering stages. For example, AS of the *NAC transcription factor 109* (*NACTF109*) during maize embryo development regulates seed dormancy by controlling ABA content in seeds (Thatcher et al., 2016). *FLOWERING LOCUS C (FLC)* is an important repressor of flowering in Arabidopsis (Andersson et al., 2008; Sharma et al., 2020), and *AtU2AF65b* is a splicing factor involved in ABA-mediated regulation of flowering time in *Arabidopsis* by splicing *FLC* pre-mRNA (Xiong et al., 2019; Lee et al., 2023). JASMONATE ZIM-DOMAIN (JAZ) is a key regulators of jasmonate (JA) signaling in plants (Yan et al., 2009). In *Arabidopsis*, the JAZ protein binds to the transcription factor MYC2 and inhibits JA signaling during quiescence. Binding to the hormone receptor *CORONATINE INSENSITIVE 1 (COI1)* upon hormone induction leads to degradation of JAZ. This degradation allows *AtMED25* to activate MYC2 and promote JA signaling. *AtMED25* regulates JAZ gene replacement splicing by recruiting splicing factors PRP39a and PRP40a, preventing excessive desensitization of JA signaling mediated by JAZ splice variants (Pauwels and Goossens, 2011; Wu et al., 2020). In rice (*Oryza Sativa*), *OsDREB2* activates the expression of downstream genes involved in heat shock stress response and tolerance. The direct homolog of *OsDREB2B* enhances the ability of plants to cope with drought stress through AS by directly producing *OsDREB2B2* by splicing I1, E2, and I2 at once under drought stress (Matsukura et al., 2010).

Different gene variants affecting alternative splicing (AS) have been observed in numerous functional gene studies. These variants play a crucial role in phenotypic changes. For instance, in poplar (*Populus tomentosa*), age-dependent AS triggers an aberrant splicing event in the pre-mRNA encoding *PtRD26*. This event leads to the production of a truncated protein, PtRD26IR, which acts as a dominant negative regulator of senescence by interacting with multiple senescence-associated NAC family transcription factors, inhibiting their DNA-binding activity (Wang et al., 2021). In *Arabidopsis*, the RNA-binding splicing factor SUPPRESSOR-OF-WHITE-APRICOT/SURP RNA-BINDING DOMAIN-CONTAINING PROTEIN1 (SWAP1) interacts with the splicing factor complexes SPLICING FACTOR FOR PHYTOCHROME SIGNALING (SFPS) and REDUCED RED LIGHT RESPONSES IN CRY1CRY2 BACKGROUND 1 (RRC1). These complexes regulate pre-mRNA splicing and induce alterations in photo morphology (Kathare et al., 2022). In bread wheat (*Triticum aestivum*), two variable splicers, Pm4b_V1 and Pm4b_V2, of the powdery mildew resistance gene Pm4b interact. In brief, Pm4b_V2 enhances wheat disease resistance by recruiting Pm4b_V1 from the cytoplasm to the endoplasmic reticulum (ER) by forming an ER-related complex (Sanchez-Martin et al., 2021).

## 2 Detection of alternative splicing using transcriptome sequencing

The continuous advancement of RNA sequencing (next generation sequencing) and long-read isoform sequencing (Iso-seq) has significantly enhanced our ability to study alternative splicing comprehensively. Two primary computational approaches have been employed to investigate splicing diversity using RNA-seq data.

Transcript reconstruction methods: These approaches focus on inferring isoform usage frequency by utilizing probabilistic models to reconstruct each isoform based on the read distribution mapped to a specific gene. Typical software packages include Cufflinks (Trapnell et al., 2010), StringTie (Pertea et al., 2015), MISO (Yarden et al., 2010), SpliceGrapher (Mark et al., 2012). Indeed, transcriptome reconstruction is an exceptionally challenging problem in the field of bioinformatics and computational biology (Estefania et al., 2021). Single-molecule long-read sequencing technology has emerged as a valuable tool in transcriptome sequencing due to its ability to generate long reads with high throughput. The utilization of Iso-seq has become a preferred approach for sequencing more comprehensive and full-length transcriptomes, enabling the prediction and validation of gene models with greater accuracy and completeness. By producing long reads that can span entire transcript isoforms, Iso-seq overcomes some of the challenges associated with transcriptome reconstruction, such as accurately detecting complex splicing events and resolving alternative isoforms that may be missed by short-read sequencing. However, they are not suitable to pinpoint splicing events but whole sequences of transcripts. For instance, degraded and immature RNA as well as DNA fragments in the RNA samples can be erroneously identified as novel genes and transcripts in the Iso-seq data. In practice, tools such as TAMA software (Sim et al., 2020) could determine splice junctions and transcription start and end sites accurately. Unfortunately, the current cost of third-generation sequencing is high, and the detection of all transcripts may be limited by the depth of sequencing and the number of samples. Therefore, the development of tools combining RNA-seq and Iso-seq could effectively solve these problems. Regrettably, no mature tools have been released so far.

The second computational approach involves utilizing junction and/or exon information to infer, annotate, and identify novel splicing events (**Table 1**). Several methods, such as rMATS (Shen et al., 2014), MAJIQ (Vaquero-Garcia et al., 2016), and LeafCutter (Li et al., 2018), utilize junction information to identify these splicing events. On the other hand, DEXSeq (Anders and Huber, 2010) specifically focuses on analyzing the differential usage of exons between different experimental conditions. Two main methodologies are commonly used to quantify alternative splicing (AS) events: the percent spliced-in (PSI) and the splicing index (SI). PSI provides an estimate of the relative usage of each alternative pathway of an AS event. In contrast, the splicing index (SI) measures the relative signal or coverage of an exon or a junction compared to the entire gene.

**Table 1:** Algorithms for the identification of Alternative Splicing events.

Algorithm	E.	S.	V.	PSI	D.	Information used for quantification	References
Aspli	✓	✓	×	✓	×	Only junctions	(Mancini et al., 2021)
Leafcutter	×	✓	✓	✓	×	Only junctions	(Li et al., 2018)
CASH	✓	✓	×	∼	✓	Exons and junctions	(Wu et al., 2018)
SplAdder	✓	✓	×	✓	✓	Exons and junctions	(Kahles et al., 2016)
SGSeq	✓	×	✓	✓	×	Exons and junctions	(Xing et al., 2016)
MAJIQ+VOILA	✓	✓	✓	✓	×	Only junctions	(Vaquero-Garcia et al., 2016)
EventPointer	✓	✓	✓	✓	×	Exons and junctions	(Romero et al., 2016)
SUPPA	✓	✓	✓	✓	×	Expression of isoforms involved in event	(Alamancos et al., 2015)
SplicingTypesAnno	✓	✓	✓	✓	×	Exons and junctions	(Sun et al., 2015)
SplicingExpress	✓	✓	✓	×	×	Expression of isoforms involved in event	(Kroll et al., 2015)
SplicePie	∼	✓	×	✓	×	Exons and junctions	(Pulyakhina et al., 2015)
Vast-Tools	✓	✓	✓	✓	×	Exons and junctions	(Irimia et al., 2014)
SpliceR	✓	×	×	×	✓	Expression of isoforms involved in event	(Vitting-Seerup et al., 2014)
rMATS	✓	✓	×	✓	✓	Exons and junctions	(Shen et al., 2014)
Gess	✓	✓	×	✓	✓	Only exons	(Ye et al., 2014)
SplicingCompass	×	✓	✓	×	×	Exons and junctions	(Aschoff et al., 2013)
ASprofile	✓	×	×	×	×	Expression of isoforms involved in event	(Florea et al., 2013)
DSGseq	×	✓	×	×	✓	Only exons	(Wang et al., 2013)
DiffSplice	✓	✓	✓	✓	✓	Exons and junctions	(Hu et al., 2013)
SpliceSeq	✓	✓	×	✓	×	Exons and junctions	(Ryan et al., 2012)
SpliceTrap	✓	✓	×	✓	×	Only exons	(Wu et al., 2011)
JuncBASE	✓	✓	×	×	×	Only junctions	(Brooks et al., 2011)
DEXseq	×	✓	×	×	✓	Only exons	(Anders and Huber, 2010)
AltAnalyze	✓	✓	✓	×	×	Exons and junctions	(Emig et al., 2010)

*There is not a peer-reviewed reference for this algorithm. E, event classification; S, this method provides statistics; V, visualization; PSI, whether the PSI is returned; D, Whether to make discrepancy detection.

✓, this algorithm provides this result; ×, this algorithm does not provide this result; ~, this algorithm does not provide this result, but it is easily computed.

In addition to detecting different AS events, it is important to directly compare direct AS differences across samples. The Cuffdiff (Cufflinks) (Trapnell et al., 2010) package can test for differential splicing between isoforms in different samples. In addition, CASH (Wu et al., 2018), DEXseq (Anders and Huber, 2010), DiffSplice (Hu et al., 2013), Gess (Ye et al., 2014), rMATS (Shen et al., 2014), SplAdder (Kahles et al., 2016) and other software can use different algorithms to detect different AS events between different samples. But unfortunately, none of these AS analysis software takes into account the existence of variants. Direct analysis at the allele-aware level cannot be achieved. Allele-aware AS analysis software is of great significance in analyzing the causes of variable AS, such as comparing the differences in AS between different genomic haplotypes.

## 3 Deep learning based alternative splicing study

Several models have been developed for predicting and identifying alternative splicing events combining deep learning approaches (**Table 2**). For example, DeepASmRNA is a convolutional neural network (CNN) model capable of identifying alternative splicing events with over 90% accuracy (Cao et al., 2022). The Deep Splicing Code model uses raw RNA sequences to classify exons based on their alternative splicing behavior and performs well in identifying splice sites and motifs (Louadi et al., 2019). The deep-learning model AbSplice predicts anomalous splicing, increasing the accuracy of traditional DNA-based anomalous splicing prediction to 48% at a 20% call rate. Furthermore, integrating RNA-Seq raises the accuracy to 60% (Wagner et al., 2023). Additionally, the deep learning based computational framework called DARTS (deep-learning augmented RNA-seq analysis of transcript splicing) utilizes deep neural networks and Bayesian hypothesis testing for identifying exons based on their sequence characteristics, attaining a more than 95% accuracy rate in recognizing alternative splicing (Zhang et al., 2019). Finally the hybrid model combining CNN, recurrent neural network, and Long Short-Term Memory (LSTM) network has a splice locus identification accuracy of 96% (Nazari et al., 2019). In summary, deep learning models for alternative splicing detection have high detection accuracy, event classification, and splice site identification.

**Table 2:** Deep learning algorithms for predicting and recognizing Alternative Splicing events.

Algorithm	Neural Network	References
AbSplice	Deep Neural Network	(Wagner et al., 2023)
CI-SpliceAI	Deep Neural Network	(Strauch et al., 2022)
Deep Splicer	Convolutional Neural Network	(Fernandez-Castillo et al., 2022)
DeepASmRNA	Convolutional Neural Network	(Cao et al., 2022)
DeepIsoFun	Deep Neural Network	(Yu et al., 2021a)
DMIL-IsoFun	Convolutional Neural Network	(Yu et al., 2021a)
LSTM_Splice	Long Short-Term Memory Network	(Regan et al., 2021)
SQUIRLS	Deep Neural Network	(Danis et al., 2021)
Deep SHAP	Deep Neural Network	(Jha et al., 2020)
ESPRNN	Recurrent Neural Network	(Lee et al., 2020)
Splice2Deep	Convolutional Neural Network	(Albaradei et al., 2020)
DARTS	Deep Neural Networks	(Zhang et al., 2019)
Deep Splicing Code	Convolutional Neural Network	(Louadi et al., 2019)
DIFFUSE	Deep Neural Network	(Chen et al., 2019)
MMSplice	Deep Neural Network	(Cheng et al., 2019)
SpliceAI	Deep Neural Network	(Jaganathan et al., 2019)
COSSMO	Long Short-Term Memory Network	(Bretschneider et al., 2018)
DeepSplice	Convolutional Neural Network	(Zhang et al., 2018)
SpliceRover	Convolutional Neural Network	(Zuallaert et al., 2018)
DeepCode	Deep Neural Network	(Xu et al., 2017)

## 4 Pan-genomics-based alternative splicing study

During the lengthy process of evolution, each plant develops unique genetic influenced by geographical and environmental factors. Consequently, the genome of a single plant can no longer fully represent all the genetic information of a species, and pan-genome of a species encompasses all the genetic information of a species and captures most of its genetic diversity and can help to explore plant genome evolution (Alonge et al., 2020; Liu et al., 2020; Long et al., 2021; Qin et al., 2021), crop molecular breeding (Tao et al., 2019; Yu et al., 2021b), and construction of genotype databases (Gui et al., 2020; Peng et al., 2020; Song et al., 2021). Similarly, the pan-transcriptome is a recalling concept of the pan-genome, which reflects the set of all transcripts of a species or an organism. The aggregation group integrating AS events from different genomes in a species can better represent the whole transcriptomes of the species and can better promote the study of AS biological processes. A tool RPVG (Sibbesen et al., 2023) was released to construct spliced pangenome graphs, to map RNA sequencing data to these graphs, and to perform haplotype-aware expression quantification of transcripts in a pantranscriptome.

## 5 Conclusions and prospects

The recent the developments of third-generation sequencing technologies and detection algorithms have led to significant advances in the study of alternative splicing. While much has been identified regarding the mechanism of alternative splicing generation and some of its functions, challenges remain in the detection of alternative splicing events without reference genomes. Using the third-generation reconstruction technology can reconstruct the AS version very well, but cannot directly determine the coordinates of the AS sites. Therefore, the algorithm combined with the second generation and the third generation sequencing technologies can solve most of such problems well. Compared with state-of-the-art methods, deep learning-based models have been used to improve the detection accuracy and the number of splicing events. Allele-aware AS analysis software is of great significance in analyzing the causes of variable AS, such as comparing the differences in AS between different genomic haplotypes. In the pan-genome context, it is of great significance to integrate different transcript information from different samples. Exploring the relationship between different alternative splicing events and mutations detected by different algorithms is of great significance for mining the influence of mutations on AS events.

## Data availability statement

The original contributions presented in the study are included in the article/supplementary material. Further inquiries can be directed to the corresponding authors.

## Author contributions

XY, JZ designed the study and methodology. FS, CH, XH, HH and JZ wrote the manuscript draft. XY performed writing-review, editing and supervision. All authors contributed to the article and approved the submitted version.
